# Detecting a Stroke-Affected Region in the Brain by Scanning with Low-Intensity Electromagnetic Waves in the Radio Frequency/Microwave Band

**DOI:** 10.3390/healthcare9091170

**Published:** 2021-09-06

**Authors:** Ibrahim El rube’, David Heatley, Mohamed Abdel-Maguid

**Affiliations:** 1 Computer Engineering Department, Taif University, Taif 21944, Saudi Arabia; Ibrahim.ah@tu.edu.sa; 2 Heatley Consulting, Ipswich IP5 3RE, UK; 3 Faculty of Science, Engineering & Social Sciences, Canterbury Christ Church University, Canterbury CT1 1QU, UK; mohamed.abdel-maguid@canterbury.ac.uk

**Keywords:** stroke detection, portable head scanner, low-intensity EM waves, intrinsically safe, low carbon footprint

## Abstract

There is a compelling need for a new form of head scanner to diagnose whether a patient is experiencing a stroke. Crucially, the scanner must be quickly and safely deployable at the site of the emergency to reduce the time between a diagnosis and treatment being commenced. That will help to improve the long-term outlook for many patients, which in turn will help to reduce the high cost of stroke to national economies. This paper describes a novel scanning method that utilises low-intensity electromagnetic waves in the radio frequency/microwave band to detect a stroke-affected region in the brain. This method has the potential to be low cost, portable, and widely deployable, and it is intrinsically safe for the patient and operator. It requires no specialist shielding or power supplies and, hence, can be rapidly deployed at the site of the emergency. That could be at the patient’s bedside within a hospital, at the patient’s home or place of work, or in a community setting such as a GP surgery or a nursing home. Results are presented from an extensive programme of scans of inanimate test subjects that are materially valid representations of a human head. These results confirm that the scanning method is indeed capable of detecting a stroke-affected region in these subjects. The significance of these results is discussed, as well as ways in which the efficacy of the scanning methodology could be further improved.

## 1. Introduction

Strokes are the 4th most prevalent cause of death and the leading cause of long-term invalidity in the UK [1]. Globally, the statistics are considerably worse with strokes being the 2nd most prevalent cause of death, although they are only the 3rd leading cause of long-term invalidity [2]. In the UK, around 110,000 people experience a stroke each year and around 1.2 M survivors are living with the consequences today. The treatment and rehabilitation for these patients, including the loss of productivity in the workplace and the high volume of benefit claims, costs the UK economy around GBP 26bn annually [1]. That figure is projected to reach GBP 75bn by 2035 if the current trajectory is sustained.

Given that the total healthcare expenditure in the UK for 2018 was GBP 214.4bn, which accounted for about 10.0% of GDP that year [3], it is clear that the cost of stroke alone is a significant percentage. That cost is intimately linked to the survivability of stroke patients and the proportion who require protracted treatment and long-term rehabilitation. The percentage figure for that proportion is influenced by the time between the occurrence of their stroke and treatment being commenced. The often-quoted mantra in medical circles, “time is brain”, perfectly sums up the criticality of stroke patients receiving treatment promptly in order to save as much healthy brain tissue as possible and lessen the long-term consequences. Stroke patients who receive treatment within the first hour following their stroke—the so called “golden hour”—have the highest probability of a good recovery requiring little, if any, rehabilitation (assuming there are no pre-existing underlying health issues that dominate the outcome). However, once past the golden hour, the long-term outlook for surviving patients begins to decline, and beyond around 3–4 h the outlook rapidly diminishes, with some degree of long-term invalidity becoming inevitable. Typically, around 66% of these patients leave hospital with a long-term disability [1].

The current pathway for stroke patients requires them to be transported from the site of the emergency, which in many cases is their own home or place of work, to the nearest acute stroke unit to receive a CT and/or MRI scan. Only then can a conclusive diagnosis be made on whether the patient has indeed experienced a stroke and about which type of stroke they experienced (ischaemic: i.e., a clot, haemorrhagic: i.e., a bleed). Only then can the appropriate treatment be administered. Delays, sometimes significant, can occur at several points in the pathway, between the emergency call being made and treatment commencing. In the UK, thrombolysis treatment for ischaemic strokes, which are about 85% of all cases [1], is only licensed to be administered to patients within 4.5 h from the onset of their symptoms [1]. If the time when symptoms began is unknown, or it is known that more than 4.5 h have elapsed since symptoms began, the treatment cannot be provided. The outlook for those patients is inevitably compromised given that thrombolysis reportedly increases the chance of a good outcome by 30% [1].

If a diagnosis can be made at the site of the emergency and the stroke is confirmed to be ischaemic, there is the potential for many more patients to fall within the eligibility window for thrombolysis if it can be administered at that location. That will help to increase the proportion of stroke survivors who require little or perhaps even no long-term care and rehabilitation. Statistics show that the number of patients who survive a stroke and are able to return to their normal lives without any added assistance increases by 2% when thrombolysis is given within 3 h [1]. Besides that being of huge benefit to those patients, it will also help to reduce the enormous cost of stroke to the nation. However, administering thrombolysis at the site of the emergency is not yet approved in the UK. Furthermore, there is not yet a widely available capability to determine the type of stroke at the site of the emergency. Trials are underway in some countries with specially adapted ambulances that contain a CT scanner to deliver a diagnostic capability for stroke at the site of the emergency [4,5]. These vehicles will always be extremely few in number due to their high cost, and hence, they will only be available to an extremely small number of cases that happen to arise in a favourable location. This resource, although of immense benefit to the few stroke patients involved, will nevertheless have a negligible impact on the national statistics for stroke.

**Figure 1:**
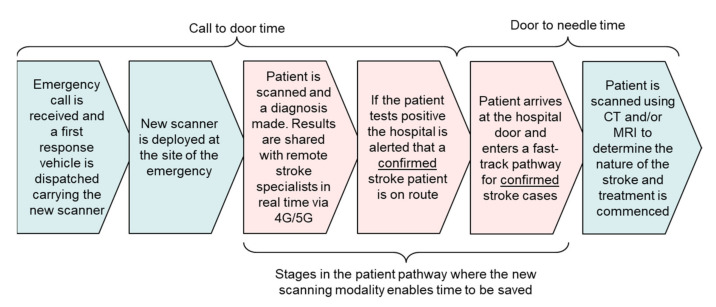
Reducing delay in the patient pathway for stroke.

However, there is also the potential for time to be saved elsewhere in the patient pathway, specifically, by shortening the door-to-needle time (i.e., the time between the patient arriving at the hospital door and treatment being commenced). Although this is not as profound a saving of time compared with commencing treatment at the site of the emergency, shortening the door-to-needle time is readily implementable within the current pathway procedures and will make an important contribution to increasing the proportion of ischaemic stroke patients who are eligible to receive thrombolysis. Figure 1 illustrates how this can be achieved by equipping the attending paramedics at the site of the emergency with a new form of head scanner that is capable of reliably determining whether the patient is or is not experiencing a stroke, regardless of the type. This is the motivation for the authors’ research reported in this paper. If the diagnosis is positive, the attending paramedics can alert the acute stroke unit’s clinicians that a confirmed case of a stroke is now in transit. In addition, diagnostic data and images could be shared with these clinicians in real time during the journey via 4G/5G mobile connections, enabling the stroke unit to be more fully prepared to fast track the patient upon arrival. To quote a seminal review of this topic published in The Lancet [6], “Stroke physicians should be engaged not only in the in-hospital phase, but also in the pre-hospital phase of acute stroke management”.

Clearly there is a compelling need and significant benefits to be gained from a new form of head scanner for stroke diagnosis that can be carried in all present-day ambulances and other first response vehicles and quickly and safely deployed at the site of the emergency. The authors are researching a new method of scanning that has the potential to meet this challenge. It uses low-intensity electromagnetic waves in the radio frequency/microwave band to detect the presence of a stroke-affected region in the brain. No specialist shielding or bespoke high-voltage power supply are required, which enables the new scanning modality to be operated almost anywhere with no prior planning. The use of low-cost COTS devices throughout the experimental apparatus and a compact, lightweight, portable construction provides a credible blueprint for a future commercially developed scanner that could be carried in ambulances and first response vehicles and operated on-scene in complete safety. Such a scanner could also be widely deployed in hospitals on crash trolleys and operated at the bedside in emergency departments and high-dependency wards, and similarly in nursing and care homes where there is a localised elderly population at an increased risk of stroke. The material and operational carbon footprint of the scanner would be intrinsically low, and the absence of any form of ionizing radiation and toxic materials avoids costly end-of-life disposal directives.

In this paper, the authors describe their research into the new scanning modality and report the latest results from a comprehensive programme of scans of inanimate test subjects that are materially valid representations of a human head. The results are presented in a visual format that illustrates how a diagnosis could be displayed to the scanner operator. This serves to highlight the simplicity in interpreting these images, which enables the operator to quickly form a diagnosis. It is clear from these results that the new modality is indeed capable of detecting the presence and location of a stroke-affected region in the test subjects. At this stage of development, it is not yet known whether the new modality has the ability to reliably determine the type of stoke—ischaemic or haemorrhagic. However, discussions with stroke specialists have revealed that the ability to reliably confirm a stroke/no-stroke diagnosis at the site of the emergency, and then to alert the acute stroke unit ahead of arrival, would be a significant and welcome advance over the current protocols. That is the focus of the authors’ current research and the results reported in this paper.

## 2. Related Work in This Field

Methods of scanning and imaging human anatomy with low-intensity radio frequencies/microwaves are being researched by other institutions across the world. For example, researchers at the University of Queensland at Brisbane, Australia [7,8,9], are investigating a scanning modality for stroke diagnosis that has some similarities with that reported in this paper. Their scanning apparatus acquires data across a similar range of frequencies, although they use a different approach to reconstruct an image of a stroke inclusion in their test subjects. They have also avoided the need for mechanical movements in their scanning apparatus by implementing a ring of stationary antennas encircling the subject that electronically translate the scanning beam in a circular path. Their results demonstrate that a stroke inclusion can be detected using their particular scanning modality, which is consistent with the findings from the different scanning modality reported in this paper.

Other institutions have taken their research in this area to commercialisation. Medfield Diagnostics (Gothenburg, Sweden) is commercialising in their Strokefinder product [10,11] with work undertaken by researchers at the Chalmers University of Technology in Gothenburg, Sweden and partner institutions [12,13]. They are also targeting stroke diagnosis using low-intensity radio/microwave frequencies; however, their approach differs from the authors of this study in several key areas. Firstly, their scanning modality uses a pulsed beam and the acquired data from the scanning chamber contains time-of-flight information, akin to radar. Secondly, their scanning beam does not translate around the phantom in a circular orbit. Instead, an array of stationary antennas is arranged in a bowl-shaped geometry that fits over the patient’s head. One antenna is assigned as the pulse transmitter at any moment while the others are receivers, then a different antenna is assigned as the transmitter while the others are receivers. That sequence progresses around all of the antennas in a defined but noncircular sequence.

Micrima (Bristol, UK) is commercialising in their Maria product [14] work that was originally undertaken at the University of Bristol [15,16]. Maria also uses low-intensity radio/microwave frequencies in a radar-like modality; however, its application is exclusively breast screening. It also uses an array of stationary antennas arranged in a bowl-shaped geometry but designed to accommodate a woman’s breast. The scanning modality and the manner in which it has been implemented in Maria affords a number of advantages over conventional breast screening, in particular, a greatly increased degree of safety for the patient and operators through the absence of X-rays, and a much-improved degree of comfort for the patient during the examination. These and other advantages are described in the referenced articles.

A common thread running through these examples and the authors’ work reported in this paper is the intrinsic safety of the scanning modalities as well as the potential for some of the scanners to be portable and deployed at the patient’s location with no prior planning. This is a profound departure from their equivalents that use X-rays or intense magnetic fields.

## 3. Materials and Methods

### 3.1. Considerations in Computed Tomography

In X-ray CT, the scanning beam is arranged to penetrate the whole subject, from front to back, then detected as it emerges on the far side. Information about the scanned subject is contained within the characteristics of the detected signal. That form of propagation and detection is labelled S21 according to scattering parameters convention (S-parameters) [17]. The extremely short wavelength of X-rays (0.01–10 nm) and the intensity of the beam ensure that the projection (i.e., shadow) cast by the subject on the detectors has a well-defined outline with little diffusion around the edges. An image of the scanned subject is reconstructed from the data delivered by the detectors using an algorithm based on the Inverse Radon Transform [18], which is well suited to the sharply defined edges of the projection and the low level of diffusion.

Initially the authors adopted the S21 configuration in their new scanning modality in deference to the well-established convention in X-ray CT. However, it was found that the Inverse Radon Transform is not suited to the new implementation because the wavelength of the scanning beam is many orders of magnitude longer than that of X-rays. In addition, the beam undergoes significantly more attenuation, diffusion, and scatter during its passage through the scanned subject. Consequently, the outline of the projection is highly blurred and feint against the naturally occurring background noise. Reconstructing an image from the acquired data is therefore significantly more challenging than the case with X-rays.

Attention is now being given to the data acquired from the reflected portion of the scanning beam, labelled S11 in the S-parameters convention. Although the scanning beam is still subjected to attenuation, diffusion, and scatter, the typically shorter path length that the reflected portion undergoes ensures that data quality is improved, particularly if the stroke-affected region in the brain happens to be close to the surface. In addition, whereas S21 requires two antennas to translate around the subject multiple times in a co-ordinated pattern, S11 requires only one antenna to orbit the subject just once. Consequently, S11 facilitates a shorter scanning duration as well as a simpler construction of scanning chamber that surrounds the subject. Details are given in the next section.

It is important to note that S11 data, although derived from the reflected portion of the scanning beam, are not the same as pulsed radar in which discrete pulses are emitted from an antenna and the reflected signals are detected. S11 data in the context of the new scanning modality derive from a continuous-wave signal—not a pulsed signal, and S11 data characterise the dielectric properties of the static environment in close proximity to the antenna—not the round-trip propagation time of pulses.

S11 data are implicit in all of the experimental results reported in this paper.

### 3.2. Experimental Scanning Apparatus

**Figure 2:**
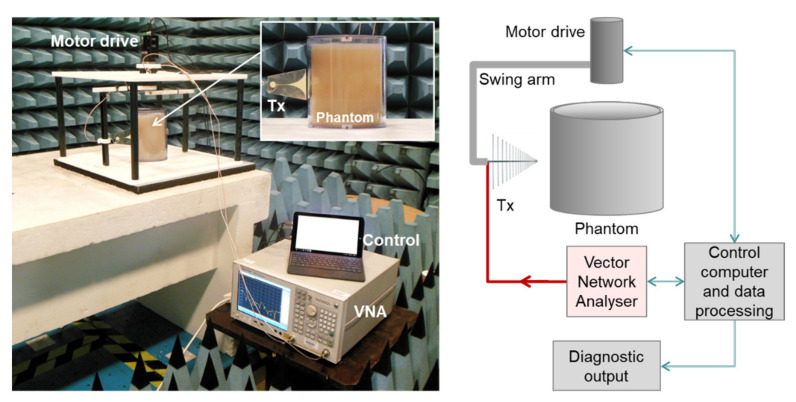
Components of the experimental scanning apparatus.

To ensure that the experimental scanning apparatus affords maximum flexibility and ease of modification, its construction employs readily available materials and devices, and a simple mechanical movement. Figure 2 shows the totality of the apparatus. It comprises a scanning chamber in which an antenna, labelled Tx, mechanically translates around the test subject, labelled phantom, under the action of a stepper motor. While the antenna is in motion, the phantom is stationary. This is the same convention used in CT. The antenna employs a compact Vivaldi design that is rated to operate across 5–18 GHz, although in reality, the operating range extends down to 1 GHz.

The antenna is connected to a Vector Network Analyser (VNA model P9374A, manufactured and supplied by Keysight Technologies, Santa Rosa, CA, USA), which measures S11 over a broad range of frequencies. The VNA, as well as the stepper motor, are under the control of a bespoke script running on a laptop PC, which also stores and processes the acquired data. It is evident that the scanning apparatus is minimalist, comprising only the scanning chamber, a VNA, and a controller. This supports the view that, in due course, a commercial version of the scanner could be compact, lightweight, and portable, as well as relatively low cost, particularly if the full-featured benchtop VNA in the photograph is replaced with a low-profile version that delivers only the required features. Preferably, the mechanically translated single antenna would also be replaced with a ring of stationary, electronically switched antennas.

During the scans reported in this paper, the antenna translates through 360 degrees in 100 equal steps (3.6 degrees per step), pausing for a brief moment at each step while the VNA measures S11 at 1601 spot frequencies between 1 GHz and 20 GHz. At each frequency, the S11 data include the magnitude and phase of the signal detected at the antenna. Consequently, each scan acquires 320,200 data points. These data and the frequency range are more than is needed for a reliable diagnosis; however, the current priority is to acquire as much data as are available to facilitate later work on refining the operation and performance of the scanner.

The use of a VNA ensures that the apparatus is highly immune to electromagnetic interference (EMI) in the surrounding environment, from sources such as Wi-Fi hubs, mobile phones, and masts, as well as other wireless services. This benefit stems from the fact that the detector side of the VNA is internally locked in frequency and phase to the transmitter side. Consequently, only the transmitted signal is recognised and accepted by the receiver. All other sources are effectively ignored. It is plausible that a future commercial development of this scanning apparatus would embed a low-profile VNA in its construction, thereby ensuring a high degree of EMI immunity. In addition, the scanning chamber that encloses the patient’s head and houses the antenna system would be designed to function as an electromagnetic screen.

A primary goal of the new scanning modality is that it must be fundamentally safe for the patient and operators and requires no specialist shielding or other safety precautions. To achieve that goal the intensity of the scanning beam must be very low. In the absence of formal regulatory guidance on the approved beam intensity for the kind of scanning modality being researched, the decision was taken early on to adopt a beam power of only 1 mW, 0 dBm. That is 100× lower than the radiated power of domestic Wi-Fi hubs (typically 100 mW, +20 dBm). At such low power levels, patients could be continuously scanned on a 24/7 basis with no safety concerns. There is no practical reason for that to be done, but it nevertheless serves to highlight the unparalleled safety margin that the new scanning modality affords compared with X-ray CT. In due course, when guidance for the new scanning modality is formally ratified by the regulatory authorities, it is reasonable to expect that the approved beam intensity will be at least 100× or even 1000× greater than the level being used because of the very short exposure period during a scan, while still remaining within the guidance limits for non-scanning wireless applications such as mobile telecommunications. However, for the time being, the authors’ research will continue with a conservative power level of 1 mW, 0 dBm. That level is implicit in all of the results reported in this paper.

### 3.3. Test Subjects (Phantoms)

**Figure 3:**
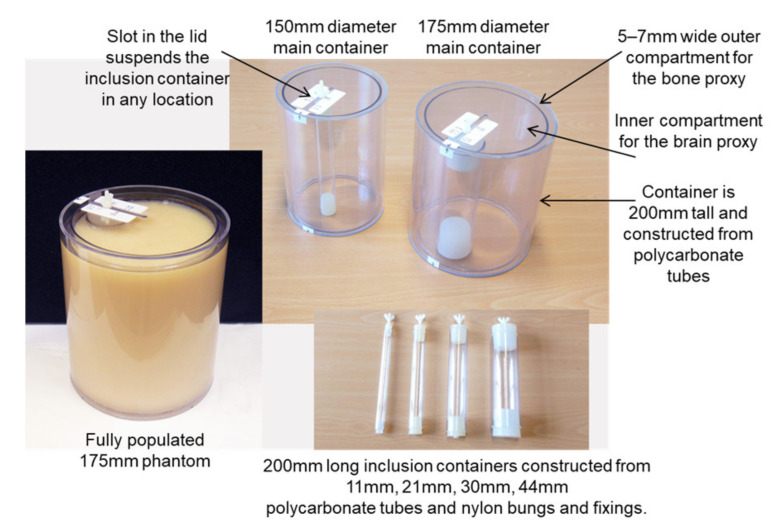
Polycarbonate vessels used in the construction of the phantoms.

The test subjects used in the scans, commonly referred to as phantoms, are constructed using fluids that closely replicate the dielectric properties of the anatomical constituents of a human head. These fluids are contained in the polycarbonate vessels shown in Figure 3. That material is used because of its high transparency at the beam frequencies. The phantoms have a cylindrical geometry in order to maintain a constant gap of 3–4 mm between the antenna and the outer edge of the phantom while the antenna is in motion around the stationary phantom. The cylindrical geometry also simplifies data interpretation by limiting the acquired data to a single slice in the horizontal X-Y plane, located at the mid-point of the vertical Z axis. Future work will use ‘head-shaped’ phantoms and will acquire data at multiple X-Y planes along the Z axis.

The 175 mm diameter container in Figure 3 represents an adult head, while the 150 mm diameter container represents an adolescent. Both containers have a 5–7 mm wide outer compartment that is filled with a proxy fluid for skull bone. The large inner compartment is filled with a proxy fluid for brain matter. Stroke-affected regions are implemented by placing one of the inclusion containers in the brain proxy fluid, anchored to the top lid of the outer container. The different diameters of the inclusion containers (11 mm, 21 mm, 30 mm, 44 mm) represent strokes of different severity and stage of progression, while their fluid contents are selected to represent an ischaemic or haemorrhagic stroke. By moving the top anchorage point of these containers along the slot in the top lid, strokes at different depths within the brain are represented. The use of nylon bungs and fixings with these containers ensures that they have minimal influence on the data acquired during scans. The photograph at the left in Figure 3 shows an example fully populated 175 mm phantom with the 44 mm stroke inclusion installed and located close to the surface of the brain. The comprehensive program of scans that produced the results reported in this paper used both phantom sizes and all four stroke inclusions at a variety of locations between the surface of the brain and the centre.

The anatomical simplicity of these phantoms contrasts with the steps taken by some of the other researchers in this field in the construction of their phantoms. For example, the University of Queensland group elected to create discrete anatomical structures within their phantoms, each with a distinct set of dielectric properties for that particular anatomical element [19]. Similarly, Micrima employed phantoms that contained a degree of anatomical geometry. Notwithstanding the undoubted validity of these approaches to phantom construction, the authors of this paper decided instead to favour an intrinsically simpler construction for several important reasons. Firstly, the wavelength of the scanning beam inside a phantom head (or a human head alike) ranges from several mm to several cm. Consequently, fine structural details within the phantom are inherently smeared out in the data, leaving just the macro-level details. It is therefore sufficient that the phantoms used in this study incorporate a simple geometry while still being a materially valid representation of a human subject. Secondly, for the purposes of a triage diagnosis at the site of the emergency, fine detail of the kind displayed in CT images is not required. The priority is firmly on determining whether the patient has or has not experienced a stroke. The simplicity of the authors’ phantoms is consistent with that priority. Thirdly, in order to physically assemble and sustain a detailed anatomical structure within a phantom, the proxy materials must have a solid, or at least a semi-solid consistency. Consequently, individual phantoms must be constructed from scratch for every different size/severity and location of the stroke-affected region that needs to be studied. That could amount to a great many phantoms if the study is wide ranging, as is the authors’ study reported herein. In contrast, the simpler anatomy favoured by the authors coupled with the use of fluid proxies enables a broad range of stroke size/severity and location to be represented with ease in just a single construction of a phantom for each head size: one for an adult and one for an adolescent.

**Figure 4:**
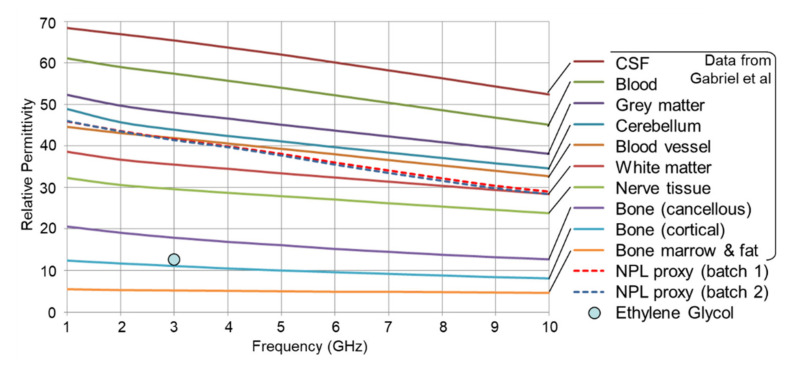
Relative permittivities of the proxy fluids obtained from NPL and published plots for other human anatomical constituents.

Sourcing the correct proxy fluids is vital for the material validity of the phantoms. The dielectric properties of the fluids, and particularly their relative permittivity as a function of frequency, define how they interact with the scanning beam of the new modality. These parameters are therefore central to selecting fluids that have a relative permittivity that is closest to the human material(s) they represent. The proxy fluid selected for brain matter is produced by the National Physical Laboratory [20] to an international standard and supplied to the telecoms industry for use in specific absorption rate (SAR) tests associated with the safety of mobile phones and the influence of their emissions on brain tissue [21]. Its relative permittivity characterises that of grey and white matter and cranial fluids (blood, CSF, ECF, ISF, etc.) in a single unified medium. This off-white opaque fluid is evident in the fully populated phantom in Figure 3. Figure 4 shows plots of its relative permittivity against frequency (measured by the supplier) and compares those plots with measured and computed plots for the individual constituents of a human body that are widely available in the literature and frequently referenced by researchers in this field [22,23,24,25,26,27]. Figure 4 also includes a single data point for Ethylene Glycol, which serves as a proxy fluid for skull bone. It too is a single unified medium that characterises cancellous and cortical bone and marrow.

The proxy fluids for an ischaemic inclusion and a haemorrhagic inclusion are RS-I fluid [28] and defibrinated sheep blood [29], respectively. Given that 85% of all diagnosed strokes are ischaemic [1], this paper focusses on the results obtained with RS-I fluid representing an ischaemic stroke. The results from defibrinated sheep blood representing a haemorrhagic stroke will be reported in due course, together with the authors’ investigation into whether the two types of stroke can be discriminated by the new scanning modality.

### 3.4. Data Processing

During a scan, the real and imaginary components of the detected S11 signal, denoted *Sr*[*tx,k*] and *Si*[*tx,k*], respectively, are acquired by the VNA at each of 100 stationary locations of the antenna *tx* as it steps around the phantom through 360 degrees. At each location, the VNA measures *Sr*[*tx,k*] and *Si*[*tx,k*] at up to 1601 spot frequencies between 1 GHz and 20 GHz, where *tx* = 1:100 is the antenna location index and *k* = 1:1601 is an index that corresponds to the spot frequencies actually used.

The signature of the stroke cannot be easily identified within this complex data for several reasons, but principally the following:

- The beam intensity launched from the antenna is very low (1 mW, 0 dBm) for the reasons given earlier. In addition, the attenuation of the beam as it propagates through the phantom is significant, particularly towards the upper end of the range of frequencies. Consequently, the signal-to-noise ratio of the acquired data is low.
- The beam undergoes significant scatter and diffusion during its passage through the phantom. This greatly reduces the definition of the signature of the stroke in the data against the naturally occurring background fluctuations and noise in the data.

To resolve these challenges several processes are performed on the dataset to facilitate a more effective search for features in the data that signify a stroke. The complex S11 signal *Sc*[*tx,k*] detected at the antenna at each measurement instant is thus expressed as follows:

(1) $$S_{c}\left[tx,k\right]=S_{r}\left[tx,k\right]+jS_{i}\left[tx,k\right]$$

**Figure 5:**
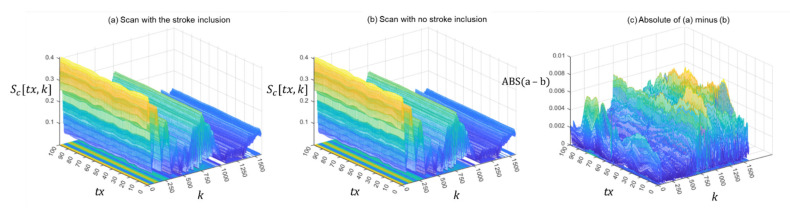
Magnitude of the raw data acquired during scan #1: (**a**) with the stroke inclusion, (**b**) without the inclusion, and (**c**) the absolute (ABS) of the difference between (**a**,**b**).

Using this expression, Figure 5a shows the magnitude of the totality of raw data acquired from the antenna during a scan of the 175 mm phantom containing a 44 mm stroke inclusion located close to the surface. The actual phantom is shown in the photo in Figure 3 in the previous section. All of the results reported in this section derive from a scan of that particular phantom, which is henceforth referred to as ‘scan #1’ for brevity. The results from scans of a broad range of phantoms and inclusions of different sizes and locations are presented in the next section.

Interestingly, when the same scan is repeated with the stroke inclusion removed from the phantom vessel, the resulting raw data in Figure 5b are superficially unchanged. This highlights the challenge faced in extracting the signature of the stroke inclusion from the raw data. It is at an extremely low level relative to the surrounding data. One potential solution explored by the authors involved subtracting the ‘no inclusion’ data from the ‘with inclusion’ data to accentuate information about the stroke inclusion and its location. This method was ultimately rejected for two primary reasons. Firstly, in a practical setting, it is all but impossible to envisage a scenario when clinicians will have two recent scans of the same patient: one taken shortly before the onset of their stroke and the other taken while their stroke is occurring. Consideration was given to utilising publicly accessible libraries of scans of healthy patients and developing a method to use those data as a generalised ‘no inclusion’ scan. However, the challenges in ensuring that these scans not only accurately represented a stroke patient prior to the onset of their stroke, but that they can also be formatted in a way that precisely replicates the output of the new scanning modality had it actually been used, were felt to be insurmountable. Secondly, subtracting the two scans from each other produces the highly complex data field in Figure 5c. Reliably identifying and extracting the low-intensity signature of the stroke inclusion from within a data field containing such extreme variability is challenging, particularly for stroke inclusions that are small in size and deeply seated within the brain. The decision was therefore taken to develop the following robust and computationally efficient method that reliably extracts the signature of a stroke inclusion in the raw data from just a single scan of the patient while they are experiencing their stroke.

**Figure 6:**
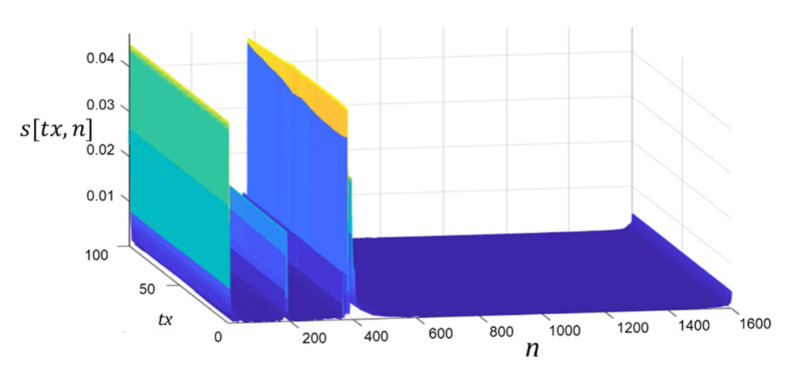
IFFT transformed data acquired from scan #1.

The Inverse Fast Fourier Transform (IFFT) is used to transform the dataset in Figure 5a from the frequency domain to the time domain. Given that the S11 scanning modality that underpins this paper uses only one antenna, the data acquired at each stationary location of the antenna as it steps around the phantom are not influenced by a second nearby antenna, as was the case in the previous S21 scanning modality that was briefly alluded to earlier. It is therefore sufficient to perform a 1D IFFT on the complex signal *Sc*[*tx,k*] in Equation (1) at each antenna location, which produces:

(2) $$s\left[tx,n\right]=\frac{1}{N}\sum_{k=1}^{N}S_{c}\left[tx,k\right]e^{j2\pi \left(n-1\right)\left(k-1\right)/N}$$

where *N* = 1:1601, *tx* = 1:100, and *n* = 1:1601. If only real data are applied to the transformation, the output data are reflected around its centre. However, for the purposes of this study, the real and imaginary components of the acquired data are applied to the transformation, which yields values in just the first half (i.e., left half) of the transform domain, as is evident in Figure 6.

The output of the IFFT, denoted *s*[*tx,n*] in Equation (2), comprises *N* complex 1D sequences that are computed independently in accordance with the structure of the data acquired during a scan. Therefore, at each antenna location as it steps around the phantom, the average of the transformed sequence over the temporal domain is calculated by averaging the sequence *s*[*tx,n*], and thus:

(3) $$s_{av}\left[tx\right]=\frac{1}{N}\sum_{n=1}^{N}s\left[tx,n\right]$$

**Figure 7:**
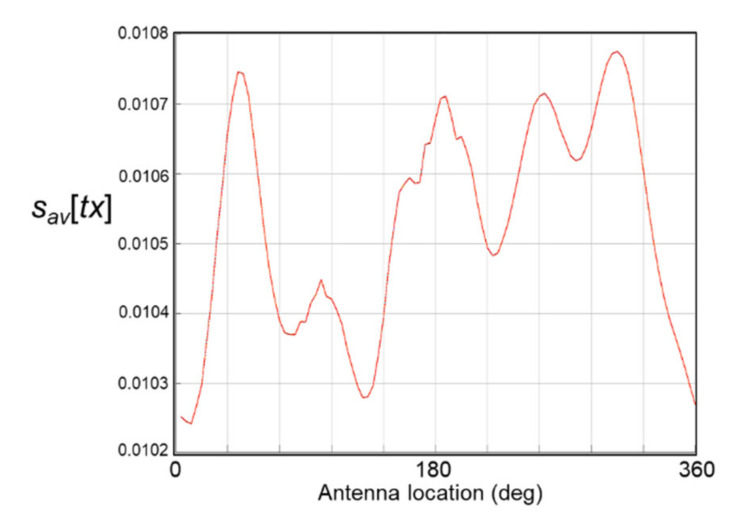
Magnitude values computed from Equation (3).

This 1D averaged data *s_av_*[*tx*] describes the S11 signal at the antenna for all frequencies that are the input to the IFFT at each antenna location. However, for each antenna location, *s_av_*[*tx*] does not yield a distinct unmistakable signature of the stroke inclusion because of the strong influence of unwanted values on it. This is evident in Figure 7, which shows multiple peaks and troughs computed from Equation (3), rather than a single distinct signature.

**Figure 8:**
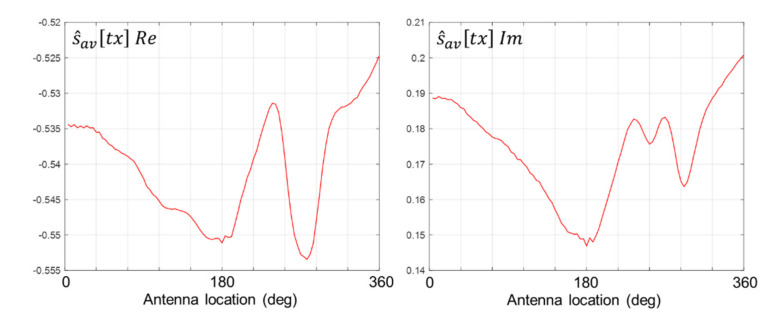
Real (**left**) and imaginary (**right**) values of ${\hat{s}}_{av}\left[tx\right]$ for scan #1.

Further study of the transformed series *s*[*tx,n*] in Figure 6 using scans of several different phantoms in which the inclusion is present in some while is absent in others, reveals that the position of *n* = *N*/4 is dominant when the inclusion is present but not when the inclusion is absent. The data sequence around the *n* = *N*/4 index can therefore be summed to resolve a more distinct signature of the stroke inclusion. Equation (3) can then be rewritten as:

(4) $${\hat{s}}_{av}\left[tx\right]=\frac{1}{2a+1}\sum_{n=\frac{N}{4}-a}^{\frac{N}{4}+a}s\left[tx,n\right]$$

where *a* ≥ 0 represents the width of the span centred on *n* = *N*/4. The value of *a* is selected in accordance with the strength of the signature of the inclusion. For example, in instances when the strength is high, the value of *a* is not critical, whereas when the strength is low, studies have found that *a* = 2 returns optimum results. Throughout the results presented in this paper, *a* is assumed to be 2. The real and imaginary components of the complex data sequence represented by Equation (4) are shown in Figure 8.

Both components carry vital information about the presence and location of the stroke inclusion. It is therefore prudent to use both. They can be combined by computing the absolute value of the averaged data sequence in Equation (4), expressed thus as:

(5) $$s_{Mag}\left[tx\right]=\left|{\hat{s}}_{av}\left[tx\right]\right|$$

**Figure 9:**
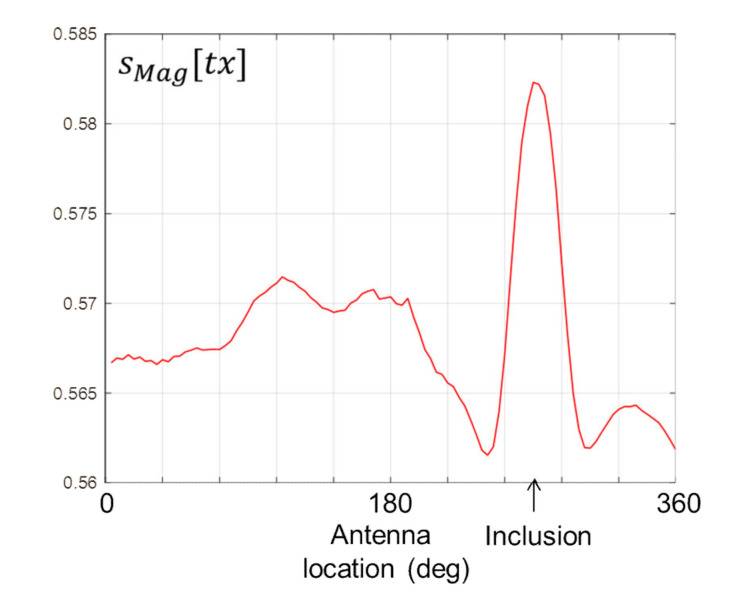
*s_Mag_*[*tx*] computed from Equation (5) for scan #1.

Figure 9 shows the data computed by Equation (5). The signature of the stroke inclusion is visible in the form of a distinctive peak, the location of which corresponds with the location of the inclusion on the horizontal axis.

To reduce the intensity of the data on either side of the peak in Figure 9, and thereby increase the distinctiveness of the signature, *s_Mag_*[*tx*] in Equation (5) can be differentiated as follows:

(6) $$s_{d}\left[tx\right]=s_{Mag}\left[tx+1\right]-s_{Mag}\left[tx\right]$$

**Figure 10:**
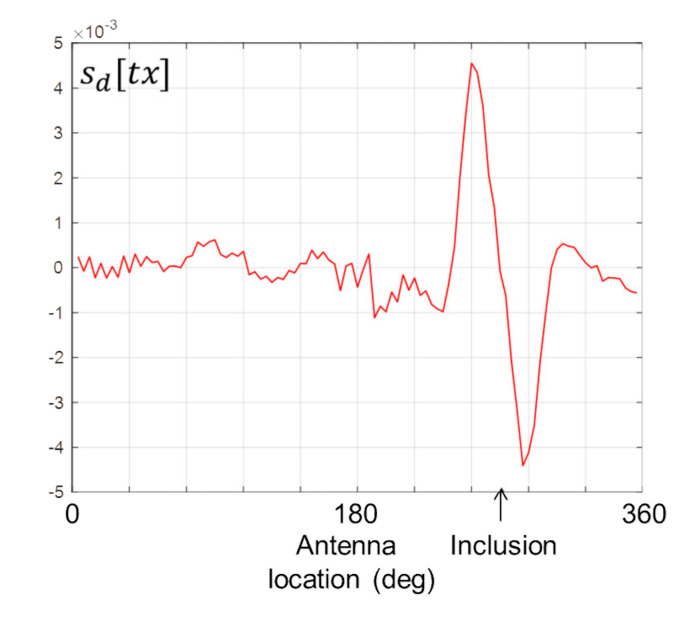
*s_d_*[*tx*] computed from Equation (6) for scan #1.

Figure 10 shows the differentiated data computed by Equation (6). The presence of the stroke inclusion is evidenced by the distinctive double peak, while the location of the inclusion on the horizontal axis coincides with the zero crossing between the two peaks.

The above results confirm that the method of data analysis devised for this study successfully extracts the signature of a stroke inclusion from the raw data acquired during a scan. The next section presents the results from a comprehensive programme of scans of phantoms of different sizes and inclusions of different sizes and locations. In this way, the results are representative of a population of adults and adolescents who are experiencing strokes of different severity and depth within the brain.

## 4. Results and Analysis

**Figure 11:**
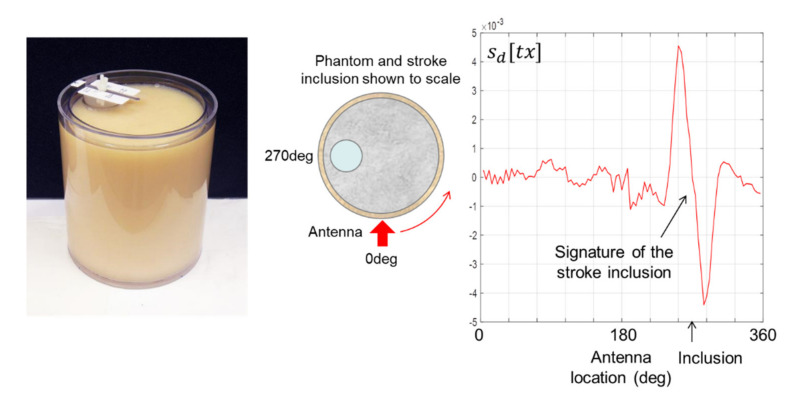
Signature of a 44 mm inclusion in the 175 mm phantom.

To ensure consistency across the scans reported in this paper, the majority were carried out with the stroke inclusion at the 9 o’clock position on a clock face, as illustrated in Figure 11 for the 44 mm inclusion in the 175 mm phantom.

The antenna begins and ends every scan at the 6 o’clock position and translates anticlockwise around the phantom. The 6 o’clock position can therefore be designated 0 degrees, as shown, in which case the inclusion is located at 270 degrees. As the antenna translates around the phantom, the signature of the stroke inclusion is highly visible as the double peak (i.e., differentiated pulse) first observed in Figure 10. The middle zero crossing between the two peaks corresponds to the location of the inclusion. This result and those that follow confirm that the new scanning modality is indeed capable of detecting the presence and location of a stroke inclusion.

**Figure 12:**
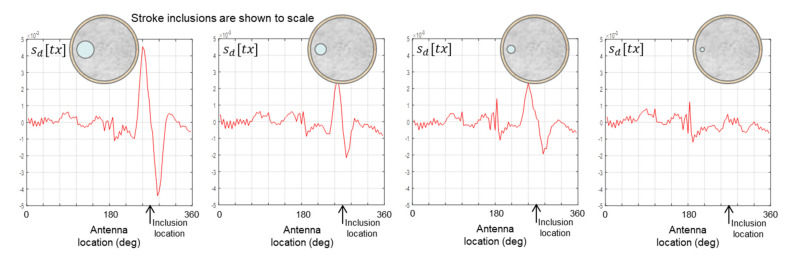
Data from scans of the 175 mm phantom with inclusion sizes of 44 mm (**left**), 30 mm, 21 mm, 11 mm (**right**).

**Figure 13:**
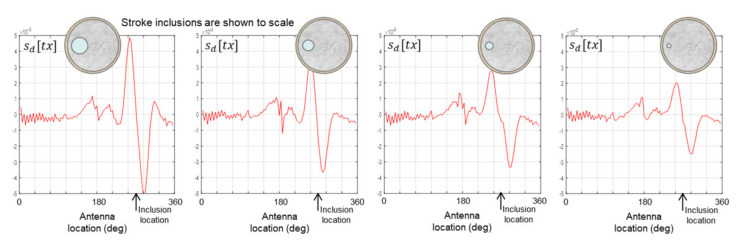
Data from scans of the 150 mm phantom with inclusion sizes of 44 mm (**left**), 30 mm, 21 mm, and 11 mm (**right**).

The data plots in Figure 12 show that progressively smaller inclusions in the 175 mm phantom are detectable down to 22 mm in size; however, the smallest 11 mm inclusion is beyond the sensitivity threshold of the apparatus. However, Figure 13 shows that the smallest 11 mm inclusion is detectable in the 150 mm phantom, which indicates that the sensitivity threshold of the apparatus is in fact at or close to 11 mm for both phantom sizes.

It is important to remember that the power level in the scanning beam is only 1 mW, 0 dBm, for the reasons outlined earlier. Had these scans been carried out at a higher beam intensity of the magnitude that could be approved by regulatory authorities in due course, it is reasonable to assume that the 11 mm inclusion, and perhaps even smaller, would be consistently detectable.

**Figure 14:**
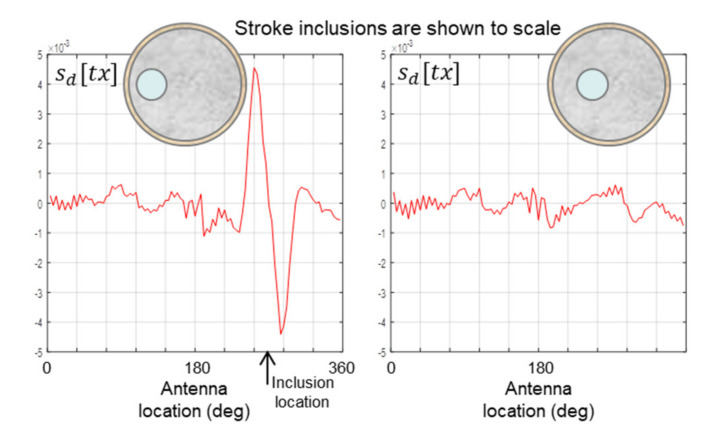
Data from scans of progressively deeper 44 mm inclusions in the 175 mm phantom.

During the scans in Figure 12 and Figure 13, the inclusion is located close to the surface of the proxy brain. The scans in Figure 14 show the impact of locating the inclusion more deeply within the proxy brain of the 175 mm phantom. Scans of the 150 mm phantom reveal the same trend, so they need not be included. It is clear that the scanning beam is unable to penetrate to a depth approximately half-way between the surface and centre of the proxy brain. Again, it is important to note the low intensity of the scanning beam and the likelihood that an approved higher intensity will penetrate more deeply and be more detectable by the apparatus.

**Figure 15:**
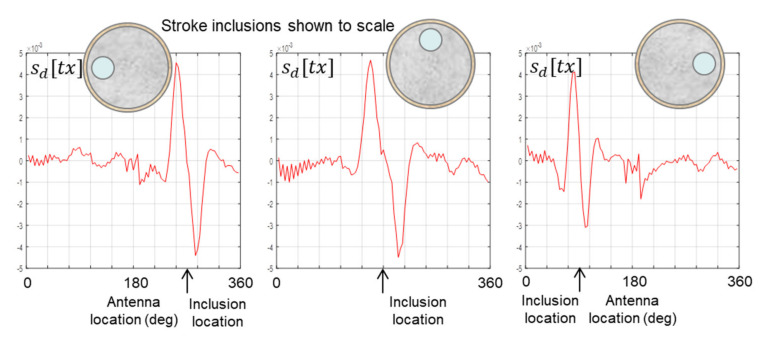
Data from scans in which the 44 mm inclusion is at different locations relative to the start of each scan.

The data plots reported thus far all derive from scans in which the stroke inclusion is located at 9 o’clock on a clock face. As stated at the start of this section, that was done to ensure consistency across those data plots and to facilitate valid comparisons between the plots. However, in order to confirm that the signature is indeed caused by the stroke inclusion and is not an artefact of the scanning apparatus or the surrounding environment that just happens to be at the correct location, the additional scans in Figure 15 were carried out with the inclusion located at 12 o’clock and 3 o’clock. The resulting data plots confirm that the location of the signature correctly tracks the actual location of the inclusion.

**Figure 16:**
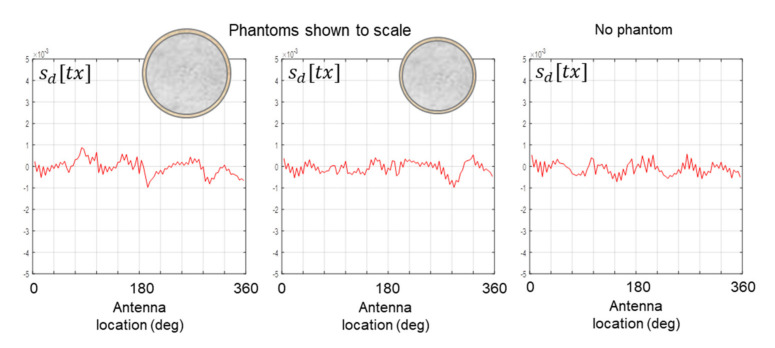
Data from scans of the 175 mm and 150 mm phantoms with no stroke inclusion present, and a scan of just the scanning apparatus.

Scans were also carried out in which there is no stroke inclusion in the phantom, as well as scans in which there is no phantom present in the scanning apparatus. The data from some of these scans are shown in Figure 16. The absence of any form of signature provides further confirmation that neither the framework of the phantom (i.e., the structural vessel excluding any inclusion) nor the scanning apparatus and the surrounding environment influenced the results reported throughout this paper.

## 5. Discussion

The results confirm that the new scanning modality is capable of detecting the presence and location of a proxy for an ischaemic stroke. The clarity of the signature of the stroke in the data is testament to the efficacy of the analytical procedure devised specifically for this application. Besides being computationally efficient, which helps to minimise the time to display a diagnosis, the simplicity of the signature it produces lends itself to rapid, unambiguous interpretation with minimal training. It should, however, be noted that the phantoms used in the scans are simplified, idealised versions of a human subject. Notwithstanding that the phantoms were constructed from proxy materials that closely replicate the dielectric properties of human tissue, fluids, and bone, the complexities of a vascular structure and the anatomy of different tissue types and fluid-filled cavities are absent in the phantoms. In justification of that, the simplification of the phantoms should be viewed as an ‘averaged’ human subject in which the boundaries between different anatomical regions are blurred to the point of completely merging into one medium. Indeed, the proxy medium used in the phantoms for brain matter is a single fluid specifically manufactured by NPL [20] to a materially valid formula that represents the unified dielectric properties of white and grey matter and all brain fluids. Furthermore, given that the wavelength of the beam inside a phantom or a human subject alike ranges from several mm to several cm, fine structural details present in the subject are inherently smeared out in the data, leaving just the macro-level details, which are manifest in the signature of the stroke. It is therefore consequential, as well as beneficial, that the phantoms used in this study need only incorporate a simple geometry while still being a valid representation of a human subject.

The long wavelength of the scanning beam also speaks to an important distinction between the new scanning modality and X-ray CT. For the purposes of a triage diagnosis at the site of the emergency, the fine detail in CT images is not required. Indeed, even the location of the stroke is not essential. The priority is firmly on determining whether the patient is or is not experiencing a stroke. The new scanning modality has demonstrated its suitability in that role. The fact that it also indicates the location of the stroke is an added benefit.

In a further simplification of the anatomy of the stroke inclusion, the authors assumed that any previous or non-vascular cerebral lesions that the patient might have experienced are closely co-located with the stroke-affected region itself. Consequently, the anomalous region that is detected is assumed to be a singular amorphous mass. However, in practical settings that assumption is not always valid. Previous or non-vascular cerebral lesions could be present in locations removed from the stroke-affected region. To take account of this, the next phase of the study will include phantoms that contain multiple inclusions to represent stroke patients whose ongoing stroke and previous cerebral lesions are dispersed throughout the brain.

Beam intensity has been shown to be a critical factor in the ability to detect a deeply seated stroke inclusion. Striking the optimum balance between having sufficient intensity to penetrate the patient’s brain to a useful depth while remaining safe to the patient and scanner operator is a fundamental objective of the new scanning methodology promoted in this paper. It is creditable that the current experimental apparatus, despite using a very low-beam intensity, is achieving sufficient penetration for stroke inclusions close to the surface to be detected, even for inclusions as small as 11 mm. However, given that the current beam intensity of 1 mW (0 dBm) is some 100× lower than that emitted by a domestic Wi-Fi hub, there is scope to increase the beam intensity by 100× or even 1000× while still remaining within the guidance limits for non-scanning applications such as mobile telecommunications. In due course, it is reasonable to expect that medical regulatory authorities will approve beam powers significantly higher than those used in this study given the very short exposure period. The next phases of the project will employ higher powers to assess the performance of the new scanning modality under more realistic conditions.

It is important to emphasise that the new scanning modality will not replace nor displace X-ray CT or MRI; quite the contrary. Its purpose is to add a valuable new capability for stroke diagnosis that complements X-ray CT and MRI in settings where they are unsuited, particularly when the scanner needs to be brought to the patient’s location.

The whole life cost is a further important consideration that favours the new scanning modality. The end-of-service disposal costs of X-ray CT and MRI scanners can be a significant proportion of their whole life cost. In contrast, the scanning modality described in this paper does not use radioactive devices nor does it produce any form of toxic long-term contamination. In addition, its energy carbon footprint is significantly lower since it does not require a specialist high-voltage power supply and, indeed, has the potential to be battery operated.

It should be noted that all of the results presented in this paper relate to an ischaemic stroke. Approximately 85% of all strokes are ischaemic [1], hence, why it was prioritised in this paper. The authors also carried out preliminary scans of phantoms with the proxy fluid for the inclusion is defibrinated blood to represent a haemorrhagic stroke. The results are very similar to those in this paper for an ischaemic stroke, which is to be expected since, as is evident in Figure 4, the relative permittivity of blood and CSF are very similar. It has yet to be determined whether the new scanning modality in its current experimental form is able to reliably distinguish between both types of stroke. Without that determination the correct treatment for the particular stroke cannot be commenced. Consequently, an on-scene diagnosis can only be a stroke or no-stroke determination. Nevertheless, as mentioned earlier, discussions with stroke specialists revealed that the ability to reliably confirm a stroke/no-stroke diagnosis at the site of the emergency, and then to alert the acute stroke unit ahead of arrival, would be a significant and welcome advance over the current protocol. Notwithstanding that trials are underway in some countries with specialist ambulances that contain a mobile CT unit [4,5] that can differentiate the two types of stroke, these units will always be extremely few in number due to their high cost and are therefore not a scalable solution. The hope is that new scanning modalities, such as the modality described in this paper, which have the potential to be carried in all ambulances and first response vehicles and used in complete safety, will pave the way for on-scene diagnosis and treatment if the ability to differentiate the two types of stroke can be developed and proven. Meeting that challenge is a priority supported by The Lancet article [6], which states, “The strategy of treatment directly at the emergency site (mobile stroke unit concept), could contribute to more efficient use of resources and reduce the time taken to instigate treatment to within 60 min—the golden hour—of the onset of the symptoms of stroke”. The authors’ work towards differentiating the two types of stroke will be reported in due course.

## 6. Conclusions

The results presented in this paper conclusively show that low-intensity electromagnetic waves in the radio frequency/microwave band can detect the presence of a stroke-affected region in a materially valid phantom of a human head. A key step in achieving that outcome is the computationally efficient method of data analysis devised for the purposes of this study, which makes the signature of a stroke inclusion highly visible in the raw data from the experimental scanning apparatus. The performance of this method will continue to be improved as the project progresses. Alternative methods that show good promise will also be investigated.

There is scope in the next development phases of the project to increase the intensity of the scanning beam used in this study by 100× or even 1000× and still remain within the safety guidelines for mobile communications and other non-scanning applications. That is certain to enable smaller and more deeply seated stroke inclusions to be detected. The results from that work will be reported in due course.

The next development phases will also employ more complex phantoms that represent patients who have multiple anomalous regions in their brain caused by previous or non-vascular cerebral lesions in addition to the stroke that is occurring at that moment.

It is certain that being able to administer stroke treatment at the site of the emergency has the potential to reduce the time expired from the occurrence of a stroke to the absolute minimum. However, for that fundamental departure from the current patient pathway to be approved by all of the relevant regulatory authorities and clinical and patient advisory groups, it is vital that the scanning methodology deployed at the site of the emergency is proven to be capable of reliably differentiating between ischaemic and haemorrhagic strokes. Achieving that with the scanning modality described in this paper in its current form has been shown to be challenging due to the very similar dielectric properties of the fluids involved in both types of stroke. However, given that this scanning modality undoubtedly has the potential to deliver a reliable stroke/no-stroke diagnosis at the site of the emergency, that alone will help to shorten the time to treatment by enabling the acute stroke unit to be alerted that a confirmed stroke patient is in transit. That patient can then be fast tracked upon arrival to shorten the door-to-needle time. That will make a valuable contribution towards minimising the overall time from the occurrence of the stroke to treatment being administered in hospital. That will be a highly beneficial interim measure for stroke patients until such a time in the future when new scanning modalities of the kind reported in this paper are able to reliably discriminate between both types of stroke, and treatment is approved to be administered at the site of the emergency.

There is no doubt that the new scanning modality has the potential to be simple and low cost to implement, and it is therefore suited to manufacture at scale. Such scanners could be carried in all first response emergency vehicles and be in situ in hospitals and acute stroke units, GP surgeries, and residential care homes. It is that kind of coverage that is needed to transform the outlook for stroke patients and have a significant positive impact on the current stroke statistics and the enormous cost of stroke to national economies.

## Data Availability

Not applicable.
